# The Clinical Review Committee: Impact of the Development of In Vitro Diagnostic Tests for SARS-CoV-2 Within RADx Tech

**DOI:** 10.1109/OJEMB.2021.3070818

**Published:** 2021-04-28

**Authors:** Matthew L. Robinson, Charlotte Gaydos, Barbara Van Der Pol, Sally McFall, Yu-Hsiang Hsieh, William Clarke, Robert L. Murphy, Lea E. Widdice, Lisa R. Hirschhorn, Richard Rothman, Chad Achenbach, Claudia Hawkins, Adam Samuta, Laura Gibson, David D. McManus, Yukari C. Manabe

**Affiliations:** 1 Division of Infectious DiseasesJohns Hopkins University School of Medicine 1500 Baltimore MD 21205 USA; 2 Division of Infectious DiseasesThe University of Alabama at Birmingham School of Medicine 9967 Birmingham AL 35233 USA; 3 Center for Innovation in Point-of-Care Technology for HIV/AIDSNorthwestern University 3270 Evanston IL 60208 USA; 4 Department of Emergency MedicineJohns Hopkins University School of Medicine 1500 Baltimore MD 21205 USA; 5 Department of PathologyJohns Hopkins School of Medicine 1500 Baltimore MD 21205 USA; 6 Division of Infectious Diseases and Institute for Global HealthNorthwestern University Feinberg School of Medicine 12244 Chicago IL 60611 USA; 7 Department of Pediatrics, Cincinnati Children's Hospital Medical CenterUniversity of Cincinnati College of Medicine Cincinnati OH 45229 USA; 8 Department of Medical Social SciencesNorthwestern University Feinberg School of Medicine Chicago IL 60611 USA; 9 Division of Infectious DiseasesNorthwestern University Feinberg School of Medicine 12244 Chicago IL 60611 USA; 10 RADx Tech Bethesda MD 20892 USA; 11 Division of Infectious DiseaseUniversity of Massachusetts Medical School 12262 Worcester MA 01655 USA; 12 Department of MedicineUniversity of Massachusetts Medical School 12262 Worcester MA 01655 USA

**Keywords:** SARS-CoV-2, COVID-19, in vitro diagnostics, point-of-care testing, Rapid Acceleration of Diagnostics program

## Abstract

The NIH Rapid Acceleration of Diagnostics (RADx^SM^) Tech Program was created to speed the development, validation, and commercialization of innovative point-of-care (POC) and home-based tests, and to improve clinical laboratory tests, that can directly detect SARS-CoV-2. Leveraging the experience of the Point-of-Care Technologies Research Network, a Clinical Review Committee (CRC) composed of clinicians, bioengineers, regulatory experts, and laboratorians was created to provide structured feedback to SARS-CoV-2 diagnostic innovators. The CRC convened 53 meetings with 49 companies offering SARS-CoV-2 tests in POC and reference laboratory formats as well as collection materials. The CRC identified common barriers to device design finalization including biosafety, workflow, result reporting, regulatory requirements, sample type, supply chain, limit of detection, lack of relevant validation data, and price-performance-use mismatch. Feedback from companies participating was positive.

## I. Introduction

The average in vitro diagnostic assay usually takes nearly 10 years to progress from proof-of-concept feasibility to full evaluation. [1] One year into the pandemic, the demand for SARS-CoV-2 testing continues to vastly exceed supply - less than 2 million SARS-CoV-2 tests are performed daily in the United States, yet 10 million tests per day will be required to safely open schools, and more will be required to interrupt widespread transmission. [2], [3] In order to accelerate the deployment of SARS-CoV-2 assays including rapid, point-of-care (POC) tests within the National Institutes of Health (NIH) Rapid Acceleration of Diagnostics (RADx^SM^) Tech Program, an understanding of each device's use case is necessary. How a device will be used informs early development, avoids costly delays and changes in design and workflow, and maximizes the public health benefit from its deployment.

Diagnostic innovators rarely have access to broad use case expertise including clinicians in multiple specialties, bioengineers, regulatory experts, laboratorians, and business leaders. The experience of the National Institutes of Health (NIH) National Institute of Biomedical Imaging and Bioengineering (NIBIB)-funded POC Technologies Research Network (POCTRN) has demonstrated that lack of early and granular feedback on intended device use case by expert users exposes novel diagnostics to subsequent development bottlenecks created by unanticipated clinical challenges, systems engineering and technical usability flaws, cumbersome workflows, and insufficient validation. [4] Companies applying to the RADx Tech opportunity were initially selected based on scientific innovation and early proof-of-concept performance data. The Clinical Review Committee (CRC) conducted assessments prior to design finalization to support accelerated development of SARS-CoV-2 diagnostics in the RADx Tech portfolio.

## II. Methods

We assembled a CRC comprised of 14 members including infectious disease, emergency medicine, ambulatory, pediatric, and adult clinicians, laboratorians, and diagnostic test and marketing experts with real-world bedside and clinical laboratory COVID-19 experience. Every RADx Tech-funded company at any stage beyond proof-of-concept was offered a 1-hour facilitated meeting with the CRC to provide structured feedback. Each company presented their one technologic approach to COVID-19 diagnosis, but also had the opportunity to discuss earlier stage technologies if time permitted. The CRC was intentional to not favor any specific diagnostic approach. The previous experience of the POCTRN in shepherding novel POC tests for sexually transmitted infections including HIV from proof-of-concept to Food and Drug Administration (FDA) approval was leveraged to provide SARS-CoV-2 diagnostic developers with the infectious disease and use case expertise necessary to understand how a test would be used for decision-making by clinicians and other end users. Such concepts of clinical usability and feasibility are as important to success as test performance and accuracy. The template for CRC meetings included a company presentation on the technology and device including workflow (15 minutes), validation data (5 minutes), proposed sample type and use case discussion (10 minutes), business development (5 minutes), proposed pre-clinical pilot study on performance (5 minutes), open discussion (15 minutes), and summary (5 minutes). Detailed written feedback and recommendations from the 60-minute CRC meeting was prepared by the Committee chairs and sent to each participating company and NIH within one week.

The original CRC concept emphasized pre-meeting preparation including completion of an intake form by the company and a preparatory conversation between committee chairs and the RADx Tech Team Lead. [5] The intake form included structured questions to characterize the detection technology, specimen type, collection modality, device operation, performance, and human factors characteristics. Based on the rapid pace of RADx Tech and to avoid duplication of effort, all pre-meeting work including the intake form was later eliminated. The written feedback of the CRC based on notes taken during the meeting evolved to include the following subsections: description of the technology emphasizing innovation or novel aspects, validation of performance (sensitivity, specificity, limit of detection, clinical sample data vs. spiked matrix), potential use cases, issues raised by the committee, business approach and pricing (cost of goods compared to estimated commercial price), and summary of the recommendations. Feedback from companies participating in CRC meetings was sought in the form of unstructured communication with the CRC coordinator. The NIBIB proposed that the CRC convene for 50 meetings. To facilitate scheduling meetings and allow a high attendance rate of CRC standing members, committee meetings were consistently scheduled at 8 am and 5 pm, allowing committee members to block their schedules. Due to the geographic dispersal of companies and teams, most meetings occurred at 5 PM Eastern Standard Time. Meetings were held over a virtual video-conferencing platform.

## III. Results

**FIG. 1.**
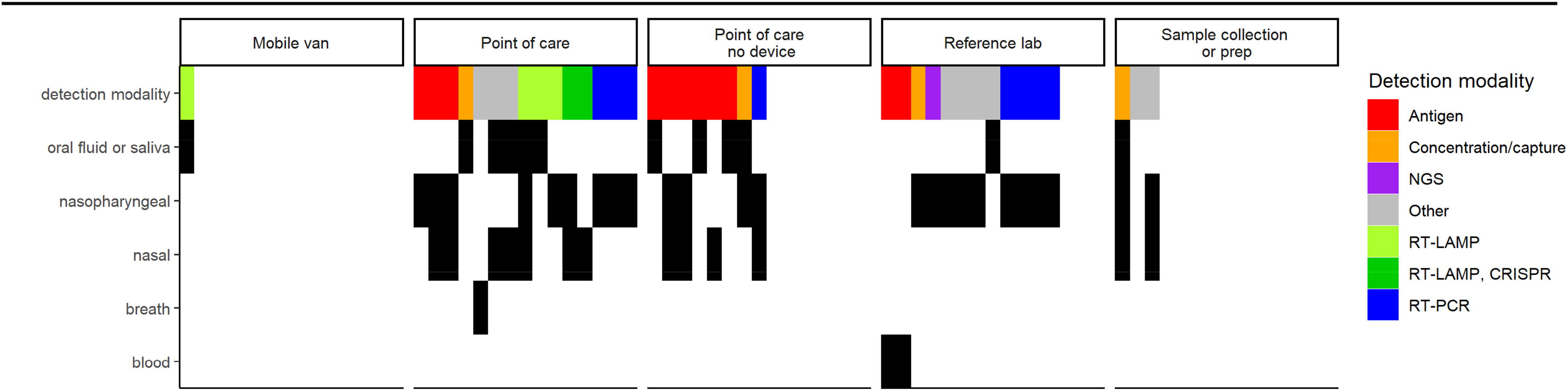
Use case, detection modality, and sample type for the reviewed technologies. Each column of a fixed width represents a single reviewed device, sorted by setting of intended use and then detection modality. Rows represent diagnostic features including detection modality followed by intended sample types.

From June 18, 2020 through December 18, 2020, 53 meetings with 49 companies occurred. The CRC reviewed devices intended for use in multiple settings including the POC (with or without a device), reference labs, mobile vans, and sample collection and preparation workflows (Fig. 1). Detection modalities included antigen detection, nucleic acid tests, concentration and capture, and others (Fig. 1). Between 4 and 12 committee members attended each meeting. Device company representatives included leadership (CEO, marketing, scientific developers, regulatory) as well as RADx Tech programmatic leadership including the Team Lead, the Portfolio Executive, FDA liaison, NIH representative, and other RADx Tech support team members.

The majority of the meeting time was used for discussion of device use workflow and suggestions for process improvement rather than use case and pre-clinical pilot studies. For the majority of assays discussed, lack of data on device performance characteristics for clinical specimens led to uncertainty around the viability of some of the devices. Common barriers to design finalization included inadequate biosafety, complex workflow, inadequate result reporting, difficulty meeting regulatory requirements, inappropriate sample types, supply chain bottlenecks, lack of optimization of the limit of detection, lack of validation data, and a mismatch of the proposed price with performance and use case (Table 1).

**TABLE 1:** Common Barriers to Design Finalization Identified By the CRC

Barrier	Examples
Biosafety/infection control	• Risk of aerosolization or spillage of infectious material, no disposal plan for infectious swab
Workflow	• Complexity of sample processing exceeds capability of operator in proposed use case
Result reporting	• No integration with lab information systems for a reference lab test, lack of mechanism for public health reporting for a home test
Regulatory requirements	• Evolving FDA requirements to achieve Emergency Use Authorization, over-the-counter considerations for Clinical Laboratory Improvement Amendments waived devices
Sample type	• Inappropriate sample type for intended use case
Supply chain	• Limited availability of proposed swab type, other consumables, or instrument
Limit of detection	• Variability in sample types, volume of buffer, input volume, efficiency of extraction and amplification
Lack of relevant validation data	• Performance characteristics reported in inappropriate matrix, overly optimistic estimation of clinical sensitivity from contrived samples
Price-performance-use mismatch	• Cost of device or consumable offers clinically insignificant improvement over existing lower cost tests, excessive cost for intended use case

Verbal summary by the facilitator at the end of the meeting detailed the points raised by committee members, areas of certainty and uncertainty, and how the company's efforts to date have the potential to address clinical and public health needs. Written summaries provided companies with detailed documentation of CRC discussion and recommendations.

Qualitative feedback from companies that participated in the CRC process included appreciation that meetings occurred early in the RADx Tech process before design finalization, perspectives represented the voice of future customers, pointed questions forced discussion of process improvement, and verbal and written meeting summaries were helpful in the detail they provided. The RADx Tech program benefited from CRC feedback as another reference point in tracking device development progress and informed the design and conduct of clinical studies performed by the RADx Tech Clinical Study Core.

## IV. Discussion

A series of joint meetings convened between 49 diagnostic companies and a CRC with relevant expertise in the clinical, laboratory, and practical challenges of infectious disease diagnostic use demonstrated a pattern of common barriers to design success. Granular questioning and feedback forced companies to confront challenges at an early development stage, prior to design finalization. The CRC was also able to highlight the innovative or distinguishing features of promising technologies. RADx Tech Team Leads learned important clinical and laboratory user views on workflow allowing them to best advise companies in their portfolios.

Optimization and feasibility testing require a clear understanding of the clinical need and the use case for the proposed assay. For example, the rapidly evolving COVID-19 field has already shown that an early reliance on nasopharyngeal swabs is being supplanted by oropharyngeal swabs [6], [7], nasal mid-turbinate swabs, sputum [8] and now saliva [9], [10] for molecular nucleic acid amplification tests as well as salivary antigen testing. [11] Self-collection versus clinician collection is also being investigated. [12] The relative sensitivity of different sample types, point of collection, severity of disease (hospitalized, ambulatory, asymptomatic) is a complicated landscape that is evolving at an unprecedented pace given the novelty of SARS-CoV-2 in aspects of its virology and clinical course of infection.

RADx-Tech funded companies originally viewed their involvement in CRC meetings as a necessary hurdle along the RADx Tech pipeline, but qualitative feedback on the sessions showed that company representatives appreciated the opportunity and challenge of considering the ‘next step of the project.’ In-depth review by the convened CRC members is a rare resource for small start-up companies; larger companies benefited from real-world, diverse viewpoints from a multidisciplinary team outside of the potentially closed perspective of industry. The CRC may serve as a model for how to quickly support clinical diagnostic assay development for other urgent diagnostic challenges in the future.

## V. Next Steps

As a next step, the CRC intends to perform assessment of the adoption and impact of specific recommendations to participating companies on their progression through RADx Tech portfolio milestones. Additional analysis of the feasibility of early risk stratification on the basis of common assessment areas will also be performed.

## VI. Conclusion

Common barriers to design finalization for SARS-CoV-2 diagnostic devices that were identified by the CRC included biosafety considerations, complicated workflows, lack of validation with real clinical specimens, and lack of clinical input on sample type and use case. Addressing risks identified by a multidisciplinary group of infectious disease and use case experts after proof-of-concept, yet early in development, has value for companies. A Clinical Review Committee when convened early in development helps companies avoid potentially costly design issues (go-no go, pivot).
